# Supplementary material for: Hypergravity Attenuates Reactivity in Primary Murine Astrocytes
Source: Biomedicines. 2022 Aug 13;10(8):1966. doi: 10.3390/biomedicines10081966 (PMC9405820; doi:10.3390/biomedicines10081966)
Supplement: Supplementary file 1 [file biomedicines-10-01966-s001.zip › biomedicines-1810993-supplementary.pdf]

Supplementary Figures

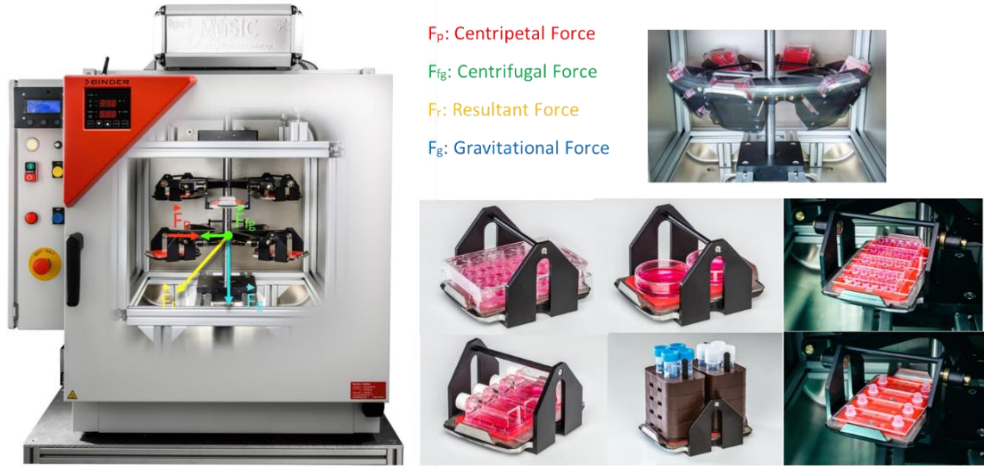

Figure S1. The Multi-Sample Incubator Centrifuge (MuSIC).

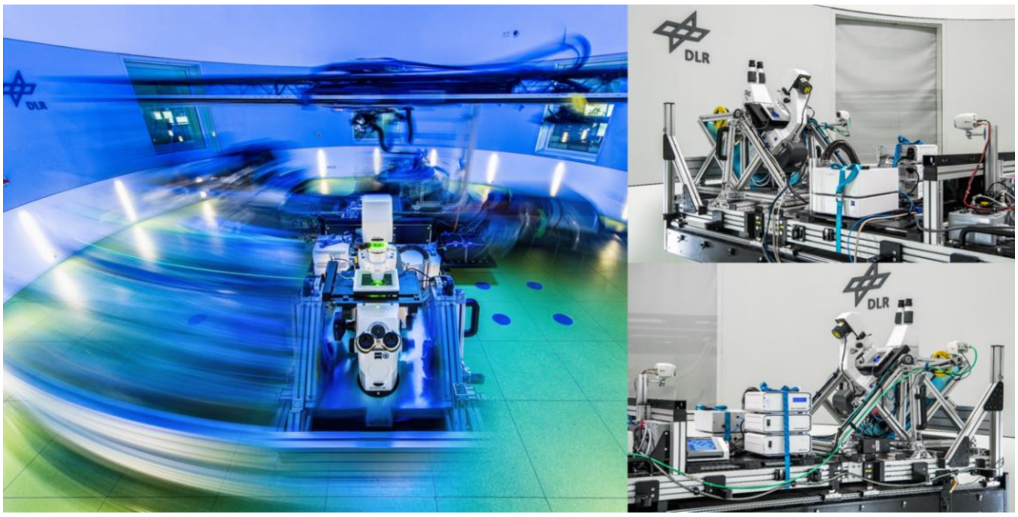

Figure S2. The Hyperscope Live-Cell Imaging Platform on the DLR Human Centrifuge.

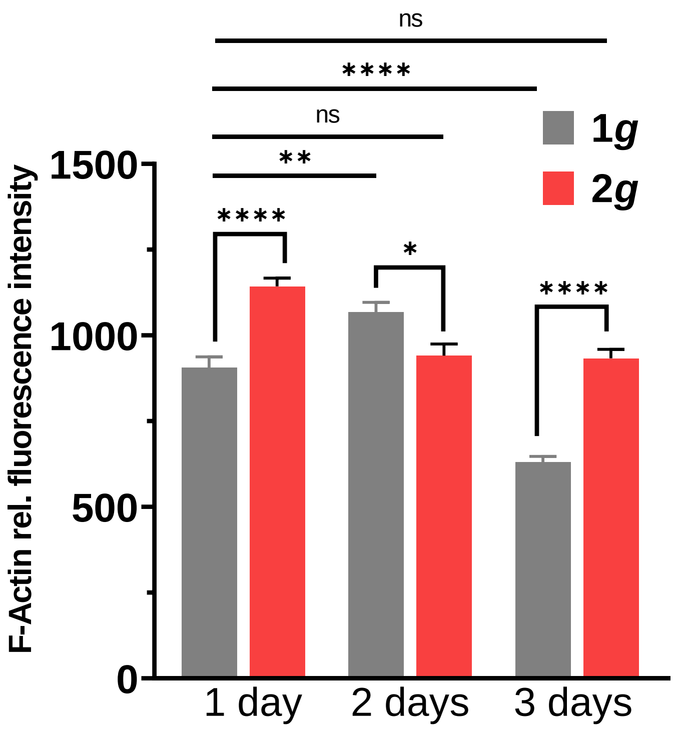

Figure S3. Phalloidin fluorescence intensity measurements under hypergravity. Quantification of fluorescence intensity values measured as mean fluorescent grey values from a Phalloidin-ATTO542 conjugate that specifically interacts with actin filaments. Mean fluorescence values were measured on astrocytes that have been exposed to 2*g* hypergravity for 1 day, 2 days, or 3 days, respectively before fixation and staining. The sample size is 350 cells derived from 2 individual astrocyte cultures from 2 gravid mice. Values are shown as SEM and were compared by *t*-test with significance indicated as follows: p>0.05 as ns, p<0.05 as *, p<0.01 as **, p<0.001 as *** and p<0.0001 as **** (2*g* 1d vs. 1*g* 1d $p < 0.0001$; 2*g* 2d vs. 1*g* 2d $p = 0.0137$; 2*g* 3d vs. 1*g* 3d $p < 0.0001$; 2*g* 2d vs. 1*g* 1d $p = 0.4626$; 1*g* 2d vs. 1*g* 1d $p = 0.0025$; 1*g* 3d vs. 1*g* 1d $p < 0.0001$; 2*g* 3d vs. 1*g* 1d $p = 0.5066$).
